# Knockout of the *Tnfa* Gene Decreases Influenza Virus-Induced Histological Reactions in Laboratory Mice

**DOI:** 10.3390/ijms25021156

**Published:** 2024-01-18

**Authors:** Darya A. Savenkova, Andrey S. Gudymo, Alexey N. Korablev, Oleg S. Taranov, Darya V. Bazovkina, Nataliya V. Danilchenko, Olga N. Perfilyeva, Elena K. Ivleva, Anastasiya A. Moiseeva, Yulia A. Bulanovich, Elena V. Roshchina, Irina A. Serova, Nariman R. Battulin, Elizabeth A. Kulikova, Dmitry V. Yudkin

**Affiliations:** 1 State Research Center of Virology and Biotechnology “Vector”, Federal Service for Surveillance on Consumer Rights Protection and Human Well-Being (FBRI SRC VB “Vector”, Rospotrebnadzor), Koltsovo 630559, Russia; savenkova_da@vector.nsc.ru (D.A.S.); gudymo_as@vector.nsc.ru (A.S.G.); korablevalexeyn@gmail.com (A.N.K.); taranov@vector.nsc.ru (O.S.T.); perfileva_on@vector.nsc.ru (O.N.P.); ivleva_ek@vector.nsc.ru (E.K.I.); chalaya_aa@vector.nsc.ru (A.A.M.); bulanovich_yua@vector.nsc.ru (Y.A.B.); minina_ev@vector.nsc.ru (E.V.R.); 2 Novosibirsk State University, Pirogova 2, Novosibirsk 630090, Russia; battulin@gmail.com; 3 Federal Research Center Institute of Cytology and Genetics, Siberian Division of the Russian Academy of Science, Lavrentieva 10, Novosibirsk 630090, Russia; daryabazovkina@gmail.com (D.V.B.); irina_serova2004@mail.ru (I.A.S.); lisa_kulikova@ngs.ru (E.A.K.)

**Keywords:** TNF-α, influenza A virus, H1N1, knockout mice, CRISPR/Cas9

## Abstract

Tumor necrosis factor alpha (TNF-α) is a cytokine that is responsible for many processes associated with immune response and inflammation. It is involved in the development of an antiviral response to many virus infections. This factor was shown to be activated in influenza A virus infection, which enhances production of other cytokines. The overexpression of these cytokines can lead to a cytokine storm. To study the role of TNF-α in the development of pathologies associated with viral infection, we generated a *Tnfa* knockout mouse strain. We demonstrated that these mice were characterized by a significant increase in the number of viral genomes compared to that in the parental strain, but the amount of live virus did not differ. A histopathology of the lungs in the genetically modified animals was significantly lower in terms of interalveolar septal infiltration. The generated model may be used to further study pathological processes in viral infections.

## 1. Introduction

Tumor necrosis factor alpha (TNF-α) is a pleiotropic cytokine known as a crucial regulator of immune responses that are involved in various inflammatory, malignant, and infectious processes. This cytokine is produced by activated macrophages, T-lymphocytes, and natural killers and exists in two different forms. The first form is a transmembrane TNF-α (tmTNF-α) that is synthesized as a 233-amino-acid precursor. Then, tmTNF-α undergoes proteolytic processing by the TNF-α-converting enzyme (TACE), which results in a soluble TNF-α (sTNF-α) form. This is a 157-amino-acid form. The part of tmTNF-α remaining in the membrane is translocated to the nucleus. Both tmTNF-α and sTNF-α exist as homotrimer proteins and provide their biological functions through binding to type 1 and 2 TNF receptors, TNFR1 and TNFR2, respectively [1,2,3,4]. TNF-α is known to activate at least three distinct effectors, which leads to the recruitment and activation of caspases, nuclear factor NF-kappa-B (NF-kappa-B), and transcription factor Jun. Therefore, TNF-a can initiate either cell death or gene expression and surviving through the interaction with its receptors [5,6].

During viral infection, TNF-α activates macrophages and dendritic cells, acts as a pyrogen in the development of fever, mediates hematopoiesis modulation, and is able to directly inhibit viral replication. Moreover, TNF-α stimulates epithelial cells to release some molecules, such as hydrogen peroxide, defensin, cathelicidin, and azurocidin, which act against various infectious agents, in particular viruses [2,7]. For example, TNF-α was found to induce secretion of β-defensin-2 by respiratory epithelial cells in response to human respiratory syncytial virus (RSV) infection, which leads to blocking RSV entry into cells [8]. TNF-α is able to inhibit human immunodeficiency virus type 1 (HIV-1) entry into cells by downregulating CD4 and CCR5 receptors on the surface of lymphoid cells. Besides, TNF-α activates expression of several factors, such as C–C motif chemokine 5 (CCL5), C–C motif chemokine 3 (CCL3), and C–C motif chemokine 4 (CCL4), which exert negative effects on HIV-1 replication [9]. Through activation of NF-kappa-B, TNF-α suppresses the tight-junction protein claudin-5 that causes capillary leakage and massive influx of plasma proteins and white blood cells infiltrating the surrounding tissue and promotes the development of acute respiratory distress syndrome (ARDS) [10,11]. However, TNF-α facilitates viral infection in some cases. Dengue virus (DENV) has been demonstrated to induce, through TNF-α expression, cytopathological changes in blood cells, endovascular dysfunction, and neurotoxicity [12,13]. HIV-1 proteins Tat, Nef, and Gp120 stimulate TNF-α expression, which facilitates viral replication and initiates the apoptosis of uninfected bystander cells. Also, the HIV-1 Tat protein interacts with TNF-α receptors, which leads to NF-kappa-B activation and promotes HIV-1 LTR activation [9,14]. 

Influenza A virus (IAV) is an enveloped virus belonging to the *Orthomyxoviridae* family and Alphainfluenzavirus genus. IAV is a negative-sense RNA virus harboring eight different genome segments [15,16]. The virus causes respiratory tract infections and initiates activation of many proinflammatory cytokines and chemokines, in particular TNF-α. In addition, TNF-α was shown to be capable of affecting susceptibility to H1N1 in humans [17,18]. In response to IAV infection overexpression of TNF-α can cause an increase in cytokine and chemokine production via activation of STAT3, MAPK, and NF-κB signaling pathways, which may lead to a cytokine storm. Uncontrolled overexpression of pro-inflammatory cytokines, such as TNF-α, can lead to destroying the endothelial barrier and cause pulmonary edema and lung injury [10,19]. TNF-α is involved in necroptosis of alveolar epithelial cells during IAV H1N1 infection. Necroptosis was shown to be associated with high morbidity and mortality of infected mice [20]. Remarkably, Tnf-α and IL-1 receptor gene knockout mice exhibited reduced morbidity and delayed mortality in infection with a lethal H5N1 virus and no such differences with the less virulent H1N1 virus [21].

To study the influence of TNF-α on respiratory tract pathogenesis associated with viral infection, *Tnfa* knockout animal models are required. In this article, we present a study of *Tnfa* knockout mice infected with a mouse-adapted H1N1 influenza virus. Our results show that Tnf-α has a negative effect on H1N1 replication, and the absence of Tnf-α can cause a decrease in lymphocyte infiltration into interalveolar septa in the early stages of infection. These findings may expand our knowledge of the role of Tnf-α in response to influenza viral infection. An understanding of Tnf-α functions during viral infection is important for the development of therapies against cytokine storm in severe infection.

## 2. Results

### 2.1. Genotypes of the Knockout Mouse Strain

**Figure 1:**
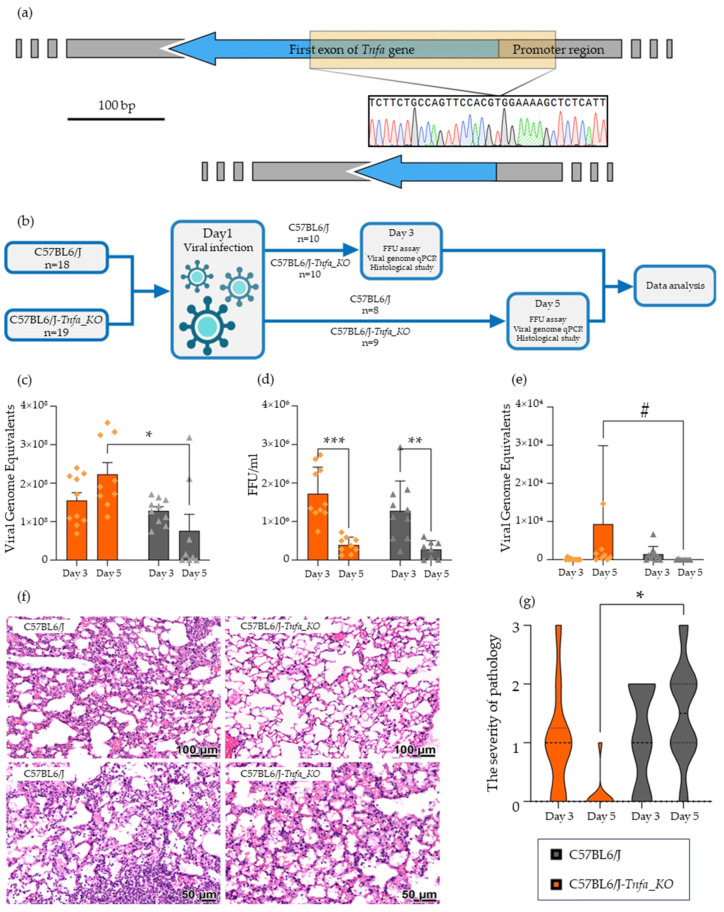
Infection of *Tnfa* knockout mice with H1N1 influenza A virus. (**a**) Scheme of deletion in *Tnfa* gene of C57BL6/J-*Tnfa_KO* mice. (**b**) Graphical representation of the experimental design. (**c**) Amount of viral genomic RNA in lungs of infected mice ^1^. (**d**) Amount of infectious viral particles in lungs of infected mice ^1^. (**e**) Amount of viral genomic RNA in brain of infected mice ^1^. (**f**) Histological alterations in organs on the 5th day after infection. Infiltration of lymphocytes into interalveolar septa is obvious in C57BL6/J mice. (**g**) Graphical representation of severity of interalveolar septal infiltration in mice ^1^. Note: * *p* < 0.05, ** *p* < 0.01, *** *p* < 0.001, # *p* < 0.1. ^1^ Legend on block (**g**) is common for blocks (**c**–**e**,**g**).

*Tnfa* knockout mice were generated using the CRISPR/Cas9 system. A 241 bp homozygous deletion (GRCm39/mm39, chr17:35420792-35421032) in the *Tnfa* gene of knockout mice was confirmed by Sanger sequencing (Figure 1a). The deleted genomic region consisted of 192 bp of the *Tnfa* first exon with the start codon and part of the *Tnfa* promoter. The analysis of the *Tnfa* mRNA expression level demonstrated the lack of *Tnfa* mRNAs in samples from C57BL6/J-*Tnfa_KO* mice.

### 2.2. Viral RNA Replication Is Enhanced in Knockout Mice

Viral genomic RNA levels in mice were evaluated using qPCR. Lung and brain samples from infected animals were collected on 3rd and 5th days after infection with H1N1 influenza virus (Figure 1b). These time points were chosen because in the previous research it was shown that peak of infection occurs in days 3–5, and the titer dramatically decreases to day 7 [22]. Here we are checking the role of Tnf-α in the presence of the virus. On the 3rd day after infection, there were no significant differences in the viral RNA level between C57BL6/J controls and C57BL6/J-*Tnfa_KO* mice in both studied organs (Figure 1c,e). On the 5th day after infection, the level of viral genomes in C57BL6/J-*Tnfa_KO* mice lungs was three-fold higher than in the controls (*p* = 0.0298). Moreover, the number of viral genomes in the lungs of C57BL6/J-*Tnfa_KO* mice increased, while that in the lungs of C57BL6/J mice decreased by the 5th day after infection (Figure 1c).

The level of viral genomic RNA in brain samples was approximately 50-fold lower than that in the lungs, regardless of the mice strain (Figure 1e). Despite the lack of significant differences (*p* > 0.05) between C57BL6/J and C57BL6/J-*Tnfa_KO* mice, there were more viral genome equivalents in the brain samples from C57BL6/J-*Tnfa_KO* mice than in those from C57BL6/J mice (*p* = 0.0590).

Next, to determine the amount of live infectious viral particles in lung and brain samples from infected mice, an FFU assay was performed. There were no significant differences in lung or brain tissues between C57BL6/J and C57BL6/J-*Tnfa_KO* mice. However, a significant decrease in infectious viral particles was detected in the lungs of both strains (C57BL6/J-*Tnfa_KO* mice, *p* = 0.0001; C57BL6/J mice, *p* = 0.0088) between the 3rd and 5th days. The mean virus count in lung samples collected on the 3rd and 5th days after infection was 1.5 × 10^6^ and 3.4 × 10^5^ FFU/mL, respectively. In the brains of infected animals, no or negligible virus counts were detected.

### 2.3. Deficiency of TNF-α Reduces Tissue Disturbance in Mice

A histological study of lung tissue was performed on the 3rd and 5th days after infection. We analyzed several parameters: interalveolar septal infiltration, perivascular and peribronchial infiltration, parenchymal consolidation, neutrophil infiltration of the alveolar lumen, intra-alveolar and intra-bronchial hemorrhages, sloughing of bronchial epithelium, sludge and separation of blood in the lung vessels, perivascular edema, alveolocyte hyperplasia, and intra-alveolar fibrin and precipitates.

Only a few of the studied parameters showed differences between C57BL6/J and C57BL6/J-*Tnfa_KO* mice. Interalveolar septal infiltration decreased in C57BL6/J-*Tnfa_KO* mice and increased in C57BL6/J mice by the 5th day (*p* = 0.0187) (Figure 1f,g). The same trend was observed for the occurrence rate of interalveolar septal infiltration (*p* = 0.0264). On the 5th day after infection, these infiltrative changes were detected in 1 out of 9 C57BL6/J-*Tnfa_KO* mice and 7 out of 8 C57BL6/J mice. Interalveolar septal infiltration was diffuse.

Perivascular infiltration was weak and tended to increase slightly by the 5th day. Peribronchial inflammatory reaction was not observed in any animal groups. Bronchial epithelium damage was present in most animals of all groups and decreased by the 5th day after infection. Disturbances of local blood circulation, such as perivascular edema, fibrin precipitates in small vessels, and intra-alveolar hemorrhages, were also observed in infected animals and increased with time.

## 3. Discussion

In the present work, we studied the role of Tnf-α in infection of laboratory mice with the H1N1 influenza virus adapted to this species. For this purpose, we generated genetically modified mice carrying a 241 bp deletion in the *Tnfa* gene, which affected part of the promoter. Also, this deletion resulted in the loss of the start codon and a frameshift. The resulting C57BL6/J-*Tnfa_KO* strain was deficient in expression of the *Tnfa* gene. Infection of these animals with mouse-adapted influenza A H1N1 virus caused a significant increase in viral genome production compared with that in the parental C57BL6/J strain on the 5th day after infection. In this case, by the 5th day after infection, the number of viral genomes decreased in the parental strain and increased in the genetically modified mice. However, the amount of live virus did not differ in both strains, regardless of the day after infection. Our findings demonstrate that *Tnfa* gene knockout in mice enhances viral genome replication, but does not increase the number of new infectious viral particles. According to previous studies, the level of live virus production is similar in Tnf-α deficient mice and the parental C57BL6/J strain, but there are no data on the changes in the number of viral genomes in tissues of infected animals [22]. An anti-Tnf-α agent, etanercept, was shown to significantly reduce the number of viral genomes. However, the influence of the drug on the influenza virus cannot be excluded [23]. It is like that the lack of cytokine Tnf-α production enhances influenza virus replication.

Histopathological analysis revealed that interalveolar septal infiltration was significantly lower in the C57BL6/J-*Tnfa_KO* strain than in the C57BL6/J strain on the 5th day after infection, but there were no differences in other indicators. The significant decrease in interalveolar septal infiltration in C57BL6/J-*Tnfa_KO* mice compared with that in C57BL6/J mice may be explained by that TNF-α is a pro-inflammatory cytokine that activates macrophages and other immune cells [1,10]. TNF-α expression was shown to induce white blood cell infiltration into surrounding tissues. Therefore, TNF-α deficiency may decrease immune cell infiltration into surrounding tissues, in particular, interalveolar septal tissue. A similar reduction in lung inflammation was revealed in mice knocked out in another cytokine, IL1B [24]. However, it is important that influenza A H1N1 infection is accompanied by the release of not only TNF-α but also other cytokines exhibiting antiviral activity, which may explain the lack of histopathological differences between the study groups in other indicators.

The influence of Tnf-α on the course of influenza A virus infection was previously studied. For example, Tnf-α receptor gene knockout mice infected with a virulent H5N1 influenza virus were characterized by low lethality; however, there was no difference between the infected transgenic mice and infected controls with a less virulent H1N1 virus [21]. Later, no difference was found in viral production between *Tnfa* knockout mice and the parental C57BL6/J strain. In this case, lung histopathology was significantly higher in the knockout strain in inflammation, injury, and remodeling [22]. These findings are partly consistent with ours. For example, we showed that live virus production did not differ in knockout mice and the original strain; however, histopathology in terms of interalveolar septal infiltration was significantly lower in the genetically modified mice than in the parental strain, and there were no differences in other histological indicators. 

It has been shown that Tnf-α is involved in inhibition of immunopathology in mouse lungs during H1N1 infection. In our study, we have demonstrated that the absence of Tnf-α can also lead to a decreasing in lymphocyte infiltration into interalveolar septa in early stage of infection. 

Therefore, the response of the generated mouse model to H1N1 influenza virus infection is different from that of the parental strain. Histopathology is reduced, while the number of viral genomes increases, which is likely due to the influence of cytokines on viral replication. The generated model may be used for further investigation of functions of various cytokines and study of pathological processes associated with viral infection.

## 4. Materials and Methods

### 4.1. CRISPR System Design and Preparation of Components for Microinjections

Single guide RNA (sgRNA) was designed with the Benchling online tool (https://benchling.com/, accessed on 22 February 2023) (Table 1) using the scoring method described previously [25]. Genotyping primers, TNF-F and TNF-R, were created using the Primer Blast tool (https://www.ncbi.nlm.nih.gov/tools/primer-blast/, accessed on 22 February 2023) (Table 1). A HiScribe™ T7 High Yield RNA Synthesis kit (NEB, Ipswich, MA, USA) and an RNA Clean & Concentrator-25 kit (Zymo Research, Irvine, CA, USA) were used for in vitro sgRNA transcription and purification, respectively.

### 4.2. Generation of Tnfa Knockout Mice

The study was conducted in accordance with the Directive 2010/63/EU of the European Parliament and of the Council of 22 September 2010 on the protection of animals used for scientific purposes and approved by the Ethics Committee of the Institute of Cytology and Genetics (protocol No. 96 of 25 October 2021). Mice were bred at the SPF vivarium of the Institute of Cytology and Genetics (Novosibirsk, Russia). After weaning, mice of the same sex were kept in groups of 4 to 5 animals per cage (Optimice, Animal Care Systems, Centennial, CO, USA) at a temperature of 24 ± 2 °C, humidity of 45–50%, and a 14:10 dark–light cycle (lights on 01:00; lights off 15:00). The mice were fed with sterile food and water ad libitum. C57BL6/J females (4–6 weeks old) and C57BL6/J males (3 months old) were used as sources of oocytes and spermatozoa, respectively. In vitro fertilization was performed according to the protocol [26]. Fertilized oocytes in a solution containing 25 ng/μL sgRNA and an equimolar concentration of Alt-R HiFi Cas9 Nuclease V3 (IDT, Coralville, IA, USA) were injected into the cytoplasm. After the microinjection, embryos were cultivated overnight, and 2-cell stage embryos were transferred into the oviducts of pseudopregnant mothers (CD-1 females). 

Born mice were genotyped for modifications with TNF-F and TNF-R primers flanking a deletion (Table 1), and one founder with a 241 bp deletion (chr17: 35420792-35421032, GRCm39/mm39) was selected for breeding and generating a *Tnfa* knockout strain. The generated mouse strain was named C57BL6/J-*Tnfa_KO*.

### 4.3. Tnfa Gene Expression Analysis

Midbrain and lung tissues were homogenized in Trizol (Thermo Fisher Scientific, Waltham, MA, USA) using a motor-driven grinder (Sigma-Aldrich, Burlington, MA, USA); total RNA was extracted according to the manufacturer’s protocol, air dried, and solved in 25 μL of DEPC-treated water. Genomic DNA traces were removed by incubation with RNAse-free DNAse (Promega, Madison, WI, USA) according to the manufacturer’s protocol. Total RNA (1 μg) was taken for cDNA synthesis using a random hexanucleotide primer mixture. The number of Tnfa and Polr2a gene transcripts was evaluated with SYBR Green real-time quantitative PCR using selective primers (Table 1) as described earlier [27,28].

### 4.4. H1N1 Infection

All animal procedures were carried out on 18 C57BL6/J and 19 *Tnfa* knockout two-month-old mice of both sexes in accordance with the European Convention for the Protection of Vertebrate Animals Used for Experimental and Other Scientific Purposes (CETS No. 123). The experimental design was approved by the Ethics Committee of the State Research Center of Virology and Biotechnology “Vector” (protocol No. 5, 31 August 2023).

Mice were inoculated with pandemic influenza virus A/California/04/2009 (H1N1)pdm09 provided by the Centers for Disease Control and Prevention (CDC, Atlanta, GE, USA) (Figure 1b). The parental virus was passaged 8 times in mice. Influenza viruses were then propagated by a single passage in 9-day-old chicken embryos. Mice were infected intranasally with 24 LD50 (5.2 lg EID50), which caused 100% mortality [29]. All procedures with live influenza virus were performed in a biosafety level 3 facility.

### 4.5. Quantification of Viral RNA Load in Biological Samples by RT-PCR

RNA was isolated using a MagnoPrime UNI kit (NextBio, Moscow, Russia). Reverse transcription was carried out using a Reverta-L kit (InterLabService, Moscow, Russia). The resulting IAV cDNA fragments were amplified using an AmpliSens Influenza virus A/B-FL kit (InterLabService, Moscow, Russia). PCR and data collection were conducted on a Rotor Gene 6000 real-time cycler (Qiagen, Venlo, The Netherlands). The calibration curve of viral RNA Ct values on the influenza virus concentration (in FFU/mL) was plotted to calculate concentrations of influenza virus strains (in FFU-equivalents/mL). The PCR assay was performed once and was considered correct when all control samples of a given test system were released [30].

### 4.6. Focus-Forming Assay

Tenfold dilutions of 10% mouse organ homogenates were prepared and added to a MDCK cell monolayer in a 96-well plate, incubated at 37 °C for 1 h, and removed. Cells were incubated in supporting medium for 18–20 h. The FFU assay was performed as described earlier [30].

### 4.7. Histological Analysis

For histological analysis, brain and lung tissue samples from both C57BL6/J and C57BL6/J-Tnfa_KO mice were fixed in a 4% paraformaldehyde solution (Sigma-Aldrich, Burlington, MA, USA) for 48 h, followed by washing in water for 10 min. The samples were sequentially dehydrated in alcohol at increasing concentrations, followed by processing in a xylene–paraffin mixture. Paraffin fixation and pathology evaluation were conducted as described previously [31].

### 4.8. Statistical Analyses

Statistical analyses were performed using the GraphPad Prism 9.2.0 software package. Data are presented as a mean and standard deviation. For normally distributed quantitative variables, the significance was determined using a two-way ANOVA test with a Tukey’s HSD test. Nonparametric Kruskal–Wallis one-way analysis of variance with Dunn’s multiple comparison technique was used to establish significant differences between nominal data or not normally distributed quantitative variables. The significance threshold for all tests was set at *p* < 0.05.

## Figures and Tables

**Table 1:** Oligonucleotide sequences used in the study.

Name	Sequence 5′-3′	Reference
sgRNA	gAGAAAGCATGATCCGCGACG(TGG)	NC_000083.7
Tnf-F	CCCTCCTAACCCGTTTTGCT	NC_000083.7
Tnf-R	TTCCTTGATGCCTGGGTGTC	NC_000083.7
Polr2a-F	TGTGACAACTCCATACAATGC	NM_001291068.1
Polr2a-R	CTCTCTTAGTGAATTTGCGTACT	NM_001291068.1
Tnf-exp-F	AGCCGATGGGTTGTACCTTG	NM_001278601.1
Tnf-exp-R	GGTTGACTTTCTCCTGGTATGAGA	NM_001278601.1

## Data Availability

All data generated or analyzed during this study are included in this published article.
